# Radiological Diagnosis and Surgical Treatment of Gallstone Ileus

**DOI:** 10.7759/cureus.38481

**Published:** 2023-05-03

**Authors:** Ethan Kosco, Myles Keener, Andrew Waack, Akash R Ranabothu, Venkatramana Vattipally

**Affiliations:** 1 Medicine, The University of Toledo College of Medicine and Life Sciences, Toledo, USA; 2 Medicine, College of Natural Sciences and Mathematics, The University of Toledo, Toledo, USA; 3 Radiology, Advanced Radiology Services, PC (Professional Corporation), Grand Rapids, USA

**Keywords:** biliary-enteric fistula, enterolithotomy, colonic gallstone ileus, small bowel surgery, computed tomography (ct ), cholelithiasis

## Abstract

We report on the diagnosis and treatment of a patient who presented with a small bowel obstruction due to gallstone ileus. This condition is an infrequent complication of cholelithiasis that presents with non-specific and intermittent findings, including bloating, early satiety, constipation, nausea, and vomiting. Contrast-enhanced CT features the classic imaging finding, called Rigler’s triad, which includes small bowel distension, gas in the gallbladder, and an ectopic gallstone. Laparoscopic enterolithotomy is employed to prevent further erosion through the gallbladder wall and into the adjacent gastrointestinal structures. The early diagnosis and treatment of gallstone ileus results in decreased morbidity and mortality.

## Introduction

Gallstone ileus is a rare small bowel obstruction that may occur as a complication of cholelithiasis [1]. It is also associated with factors such as inflammatory bowel disease, age, and diet. Unfortunately, gallstone ileus presents with vague symptoms such as constipation, nausea, and hypoactive bowel sounds, making its rare diagnosis difficult. Furthermore, delayed diagnosis can result in erosion of the gallbladder, duodenum, and colon.

Rigler's triad is the hallmark of gallstone ileus. It consists of pneumobilia, small bowel dilation, and an ectopic gallstone [2]. Although this feature can be detected using plain radiographs, Contrast-enhanced CT is considered the gold standard imaging modality [3]. When diagnosed, gallstone ileus is treated surgically with several different effective approaches [4].

## Case presentation

A 51-year-old female presented with a worsening three-day history of nausea, vomiting, and abdominal pain. She reported several prior incidences of nausea and abdominal pain. Her pertinent prior medical history was positive for hyperlipidemia, obesity, and non-alcoholic fatty liver disease. The patient reported no surgical history.

Physical exam revealed abdominal distension and hypoactive bowel sounds in all four quadrants with no guarding or rigidity. Complete blood count (CBC) and metabolic panel are delineated in Tables 1-2.

**Table 1:** Complete blood count (CBC) of the patient. “H” indicates the value is high relative to normal and “L” indicates the value is low. All other findings of the CBC were within normal limits.

WBC (x1000/ul)	Neutrophil %	Lymphocytes relative (%)	Eosinophils relative (%)	Neutrophils absolute (x1000/ul)	Lymphocytes absolute (x1000/ul)
14.42 (H)	86.3 (H)	7.6 (L)	0.5 (L)	12.44 (H)	1.09 (L)

**Table 2:** Metabolic panel of the patient. “H” indicates the value is high relative to normal and “L” indicates the value is low. All other findings of the metabolic panel were within normal limits. BUN: blood urea nitrogen; ALT: alanine transaminase; AST: aspartate transferase; ALP: alkaline phosphatase.

Chloride (mEq/L)	Carbon dioxide (mEq/L)	Blood Glucose (mg/dL)	BUN (mg/dL)	Creatinine (mg/dL)	ALT (U/L)	AST (U/L)	ALP (U/L)	Total protein (g/dL)
88.0 (L)	29.0 (H)	162.0 (H)	45.0 (H)	1.50 (H)	59.0 (H)	68.0 (H)	141.0 (H)	9.0 (H)

**Figure 1:**
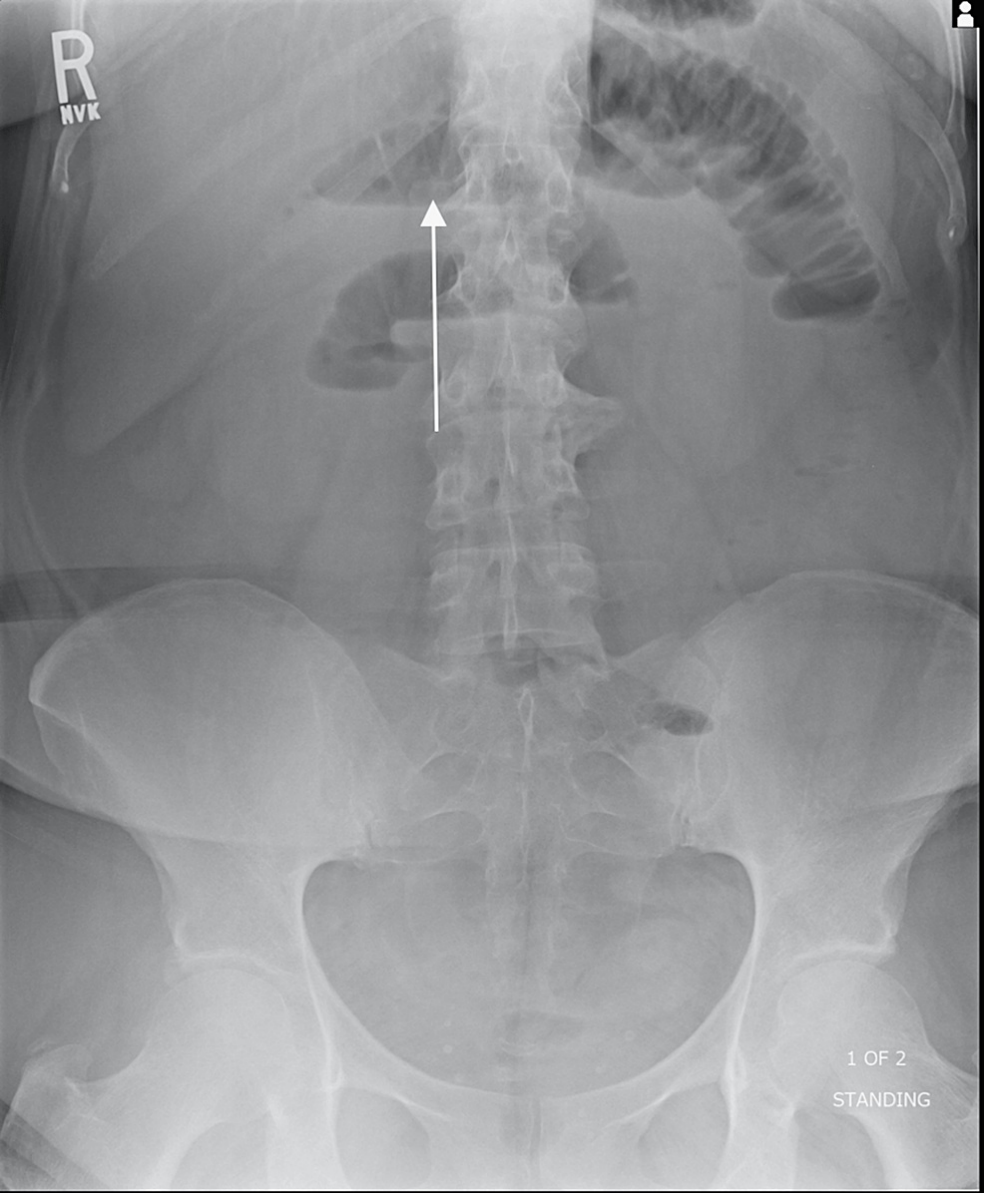
Abdominal radiograph of the upright frontal view of the abdomen demonstrating dilated loops of small bowel with air-fluid levels (arrow) and absence of gas in the colon.

**Figure 2:**
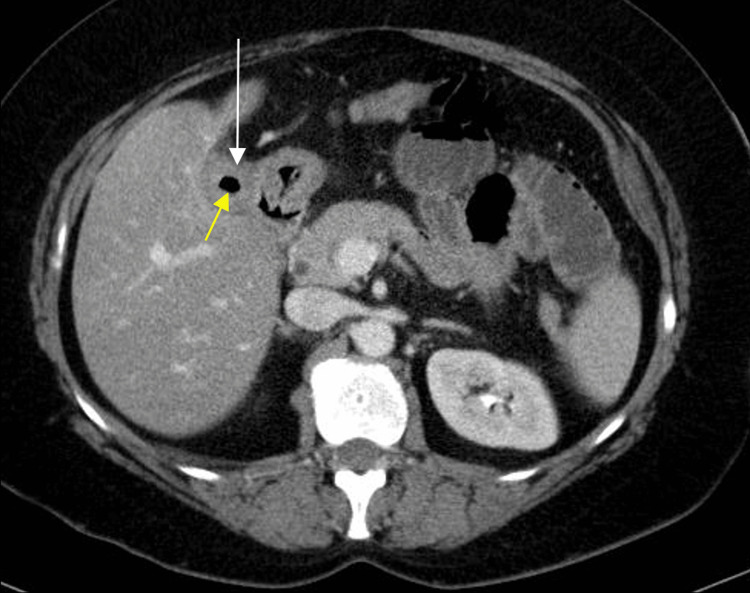
Contrast-enhanced CT abdomen and pelvis through the axial view demonstrating gallbladder wall thickening (white arrow) with gas within the gallbladder lumen (yellow arrow).

**Figure 3:**
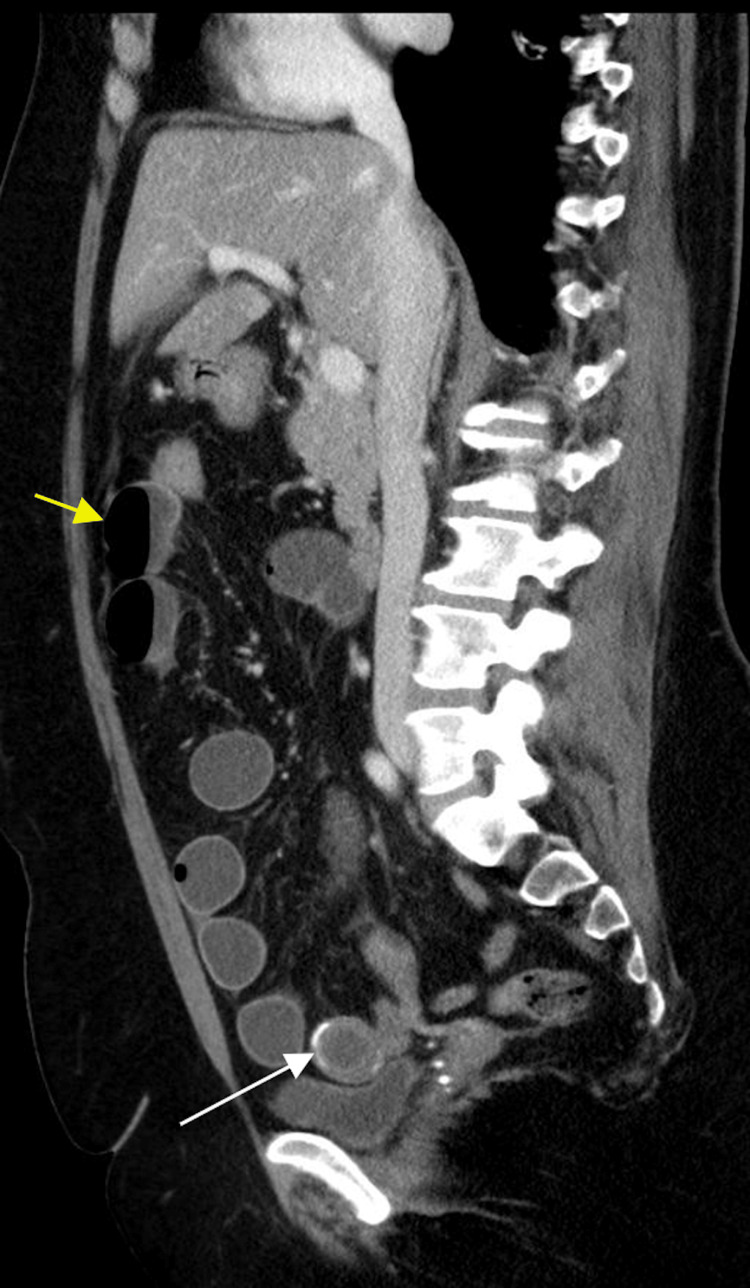
Contrast-enhanced CT of the sagittal view demonstrating a peripherally calcified gallstone (white arrow) within the terminal ileum and proximally dilated loops of the small bowel (yellow arrow) with air-fluid levels.

**Figure 4:**
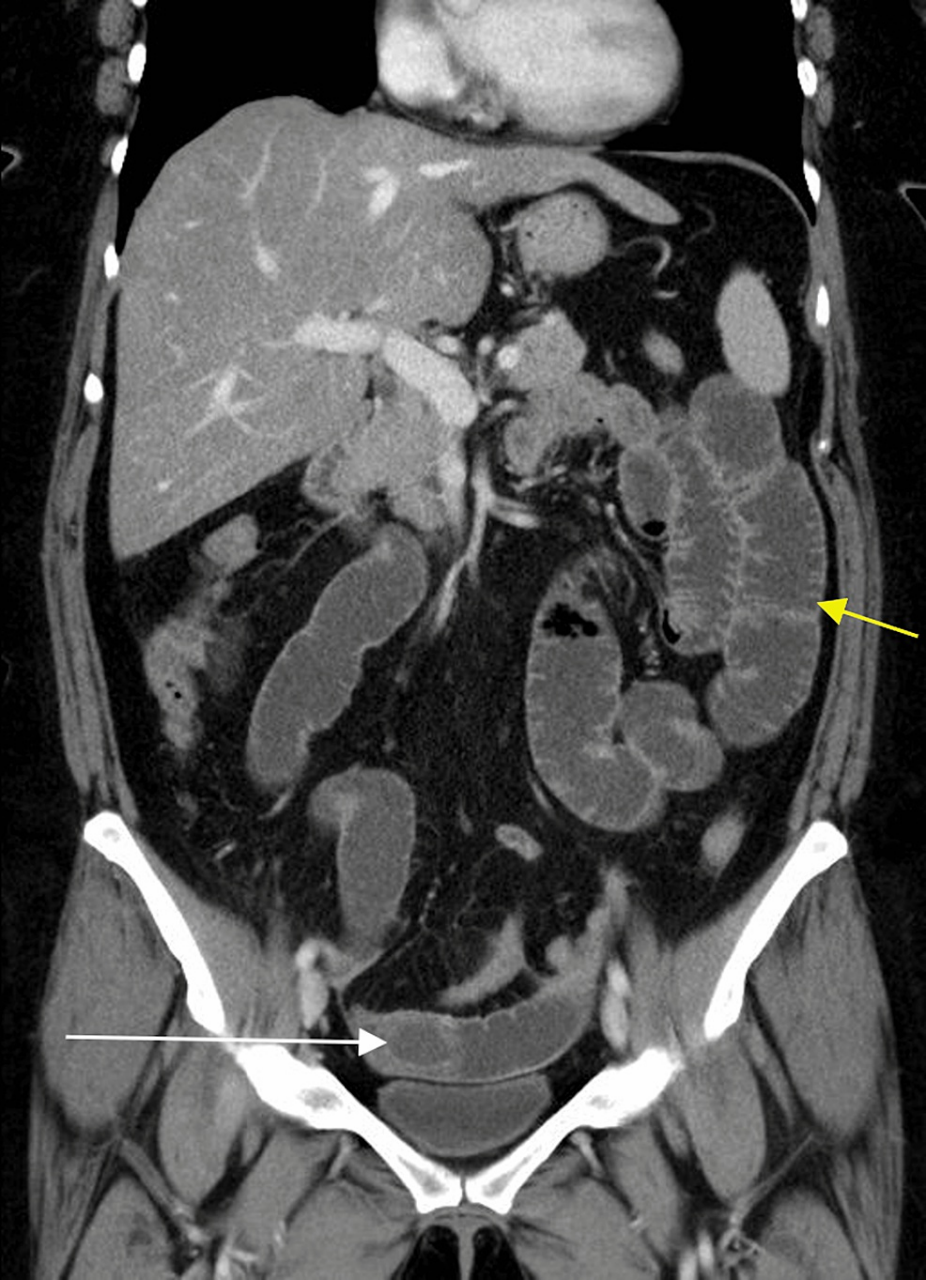
Contrast-enhanced CT of the coronal view demonstrating an obstructing mass in the distal ileum (white arrow) with proximally dilated small bowel (yellow arrow). The mass has the typical appearance of a gallstone.

Radiograph of the frontal abdomen demonstrated moderate dilation of the small bowel (Figure 1). Further contrast-enhanced CT imaging in the axial, sagittal, and coronal view displayed gallbladder wall thickening, gas buildup in the gallbladder lumen, a proximally-dilated small bowel, and an obstruction within the terminal ileum (Figures 2-4). It was hypothesized that a band of tissue was causing the obstruction. The patient agreed to immediate surgery to prevent possible systemic complications (hernia, deep vein thrombosis (DVT), bleeding, infection, injury to abdominal structures).

The patient was taken to the operating room for laparoscopic resection. Two laparoscopes were placed, one above the umbilicus and the other below the umbilicus, slightly left of the midline. The intra-abdominal cavity was clear of inflammation, and the small bowel dilations were the only abnormal findings present. While running the small bowel, a moveable mass effect was found near the ileum. To remove the mass using mini-laparotomy, the mass was milked closer to the umbilicus, and the fascial and skin incision was extended to remove a 4 cm. x 2.5 cm. cylindrical gallstone. The incisions in the bowel were closed and running the rest of the bowel did not reveal any abnormalities.

To begin removing the gall bladder and a fistula in the duodenum (whose presence was confirmed using intraoperative esophagogastroduodenoscopy (EGD)), the tissue adherent to the gall bladder was first dissected using sharp and blunt dissection. Once the fistula became visible, a GIA60 was used to cut across the healthy tissue of the duodenum and repair the abnormality. The staple line appeared stable, so the inflamed gall bladder was further dissected using hook cautery and blunt dissection. The cystic duct of the gall bladder was identified, and a clip was placed between it and the Hartmann’s pouch. Intraoperative cholangiogram did not show any intrinsic or extrinsic defect, so three clips were placed on the cystic duct stump, one clip was placed distally, and the duct was transected. The artery was also clipped proximally and distally before transecting.

The gall bladder was dissected from the liver and removed using electrocautery. The appendix was also removed by placing Vicryl ties around the base and transecting between them. After cauterization and thorough irrigation, the small bowel and colon were closely inspected for any additional masses or abnormalities. Gelfoam and a 7JP were placed in the gall bladder fossa and secured with 3-0 nylon.

## Discussion

Gallstone ileus is an uncommon etiology of small bowel obstruction, making up less than 4% of cases [4]. The most prominent determining factor for obstruction includes the size of both the stone and the bowel lumen diameter. Stones less than 2.5 cm can usually pass through into the colon and get excreted into the feces [5]. Although the majority of cases of gallstone ileus occur at the terminal ileum, due to a narrow lumen and less peristalsis activity, the ileum can occur anywhere along the small bowel [5,6]. Other determining factors vary widely depending on comorbid conditions (e.g., inflammatory bowel disease causing lumen narrowing), diet, age, etc.

The mechanism by which a gallstone ileus is created is due to a preexisting cholelith in the gallbladder which, due to pressure and inflammation [4], erodes through the gallbladder wall and into the adjacent gastrointestinal structure. The proximal duodenum is most commonly affected, but the stomach, transverse colon, and other parts of the small bowel [5] are also at risk for biliary-enteric fistula formation. Less common ways of entering the small bowel include travel through the common bile duct or a dilated ampulla of Vater [7]. The cholelith travels through the small bowel until it reaches the ileocecal junction, for which it is usually too large to pass further into the colon, becoming lodged and creating a point of obstruction. Although a large proportion of cases are due to cholecystitis, ones that are not often lead to delayed diagnosis and later clinical presentations [8]. Among cases that are not due to untreated cholecystitis and that have been reported in the literature include post-endoscopic retrograde cholangiopancreatography (ERCP) (with unsuccessful gallstone extraction) and more rarely following a laparoscopic cholecystectomy where the free cholelith erodes into the small bowel over time [9].

The symptoms of a gallstone ileus can be attributed to both the intestinal obstruction and the underlying causative factor in ileus formation (e.g., cholecystitis). Common presenting symptoms of bowel obstruction are nonspecific feelings of bloatedness, early satiety, constipation, nausea, and vomiting [10]. Signs and symptoms of cholecystitis include similar symptoms plus colicky upper quadrant abdominal pain, typically worsened after food consumption (especially meals high in lipid concentration), with or without accompanying jaundice. A physical exam may reveal high-pitched bowel sounds and abdominal tenderness [4]. The nonspecific and intermittent symptoms of gallstone ileus make diagnosis difficult. As a result, gallstone ileus continues to be associated with relatively high morbidity and mortality rates.

A thorough history can lead physicians to suspect intestinal obstruction or elucidate further gallbladder pathology workup. A comprehensive abdominal exam can further point to a specific etiology. The initial workup includes labs and imaging. Laboratory findings can help clinicians determine if a gallstone is present in the common bile duct, which would reveal an elevation of liver enzymes and bilirubin. However, these lab tests are nonspecific and usually do not help the clinician identify if or where an obstruction is occurring. Ultrasound remains the imaging modality of choice for initial suspicion of right upper quadrant (RUQ) pain, which can reveal choleliths, fistula formation, perforation, etc. [10], but can also be difficult to visualize if the dilated loops of bowel and/or gas in the bowel obscure/interfere with the imaging of the gallbladder. The sensitivity for ultrasound is reported to be 74% [11].

Radiography is typically the first imaging modality ordered for emergent gastrointestinal complaints. The combination of small bowel distension and lack of air in the colon is suggestive of an intestinal blockage. Plain radiographs (X-ray) of the chest and abdomen can identify Rigler’s triad, a radiological term for gallstone ileus which consists of pneumobilia, small bowel dilation, and an ectopic gallstone [11]. However, the sensitivity of Rigler’s triad is reported to be between 40%-70% [10]. CT shows Rigler’s triad as well, however, it is more sensitive (93%) and specific (100%) [12], and thus considered the gold standard for diagnosing gallstone ileus. The degree of calcification and size of the stone are important factors in identifying a gallstone. Because it is reported that 10% of gallstones calcify [13], it is often difficult for a radiologist to identify a causative obstructing stone. Nakao et al. reported that most stones that become obstructed are 4.3 cm in size. The average gallstone causing ileus is 4.3 cm [14] with larger ones being easier to identify. Note that it is important for the radiologist to identify free fluid and/or air as this can point to a more serious disease process that needs to be urgently identified and treated.

Gallstone ileus is treated surgically. There are three surgical approaches. The surgical approach is variable, dependent largely upon the individual patient’s general health. There are currently three approaches: isolated enterolithotomy; “one-step” enterolithotomy, cholecystectomy, and fistula closure; and a “two-step” approach, in which an initial enterolithotomy is followed by a subsequent cholecystectomy and fistula closure. The different approaches provide different risks and benefits, and the optimal technique for each individual patient is left to the surgeon’s discretion [4].

## Conclusions

Gallstone ileus is an infrequent cause of small bowel obstruction that is often associated with inflammatory bowel disease, diet, age, and cholelithiasis. Its nonspecific findings such as constipation, nausea, abdominal distension, and hypoactive bowel sounds often delay diagnosis, which contributes to high rates of morbidity and mortality. However, imaging findings such as pneumobilia, bowel distension, and peritoneal fluid on ultrasonography suggest the presence of gallstone ileus. CT imaging provides a definitive diagnosis by presenting small bowel distension, gas in the gallbladder, and an ectopic gallstone (Rigler’s triad). These early findings can prompt immediate surgical removal of the obstruction using laparoscopic enterolithotomy, which will prevent gradual erosion of the gallbladder wall, duodenum, and transverse colon. Furthermore, immediate treatment will preclude the development of a biliary-enteric fistula.
